# Engraftment syndrome after PD-1 blockade in classical Hodgkin lymphoma treated by autologous stem cell transplantation

**DOI:** 10.1007/s12185-025-04094-x

**Published:** 2025-10-31

**Authors:** Huiyeon Choi, Takuji Yamauchi, Yasuo Mori, Chie Matsuo, Hakuei Nishihara, Hidetoshi Irifune, Fumihiko Nakao, Daisuke Ishihara, Hiroshi Imanaga, Kensuke Sasaki, Teppei Sakoda, Fumiaki Jinnouchi, Kohta Miyawaki, Takahiro Shima, Yoshikane Kikushige, Koichi Akashi, Koji Kato

**Affiliations:** Department of Medicine and Biosystemic Science, Kyushu University Graduate School of Medical Sciences, Fukuoka, 812-8582 Japan

**Keywords:** Classical Hodgkin lymphoma, Checkpoint inhibitor, PD-1 blockade, Engraftment syndrome, Autologous stem cell transplantation

## Abstract

Immune checkpoint inhibitors (CPIs) targeting PD-1, such as nivolumab and pembrolizumab, have become integral to the treatment of relapsed or refractory classical Hodgkin lymphoma (cHL), frequently serving as a bridge to autologous stem cell transplantation (ASCT). However, recent reports suggest that CPI exposure prior to ASCT may increase the risk of engraftment syndrome (ES), an inflammatory complication that emerges during neutrophil recovery. We present three cases of cHL treated by ASCT was performed following anti PD-1-based salvage therapy. Two of the three patients developed ES, with one case requiring high-dose corticosteroid pulse therapy due to severe respiratory symptoms. We examined lymphocyte counts and the interval between the final CPI dose and transplantation as potential contributing factors. While lymphocyte counts did not correlate with ES severity, the patient with the highest ES severity had the shortest CPI-to-transplant interval. These findings suggest that recent CPI exposure may contribute to ES, possibly via prolonged immune activation. As CPIs become more widely used in pre-ASCT regimens, clinicians should remain alert to the potential for delayed inflammatory toxicities and consider the timing of transplantation when planning post-CPI management strategies.

**Supplementary Information:**

The online version contains supplementary material available at 10.1007/s12185-025-04094-x.

## Introduction

Immune checkpoint inhibitors (CPIs) targeting programmed cell death-1 (PD-1), such as nivolumab and pembrolizumab, have demonstrated remarkable efficacy in relapsed or refractory classical Hodgkin lymphoma (cHL) [1–3]. They are increasingly utilized as effective salvage therapies for patients with poor responses to conventional chemotherapy, often enabling successful transition to autologous stem cell transplantation (ASCT) [4–6]. However, the use of PD-1 blockade in the peri-transplant setting has raised safety concerns.

In the context of allogeneic stem cell transplantation, prior exposure to CPI is associated with increased risks of severe graft-versus-host disease (GVHD) and immune-related complications [7–9]. Similarly, CPI administered before CAR-T therapy have been linked to higher incidences of cytokine release syndrome (CRS) and immune effector cell-associated neurotoxicity syndrome (ICANS) [10–12]. These observations suggest that CPI may lead to long-lasting immune activation, which could potentially exacerbate transplant-related toxicities, even in the autologous setting.

Recent reports have highlighted a possible association between prior CPI exposure and an increased incidence of engraftment syndrome (ES) following ASCT [13, 14]. ES is a non-infectious inflammatory complication that typically occurs during neutrophil recovery and may present with fever, rash, capillary leak, or respiratory distress. Although historically uncommon in lymphoma patients, several studies have described unusually high rates of ES in cHL patients undergoing ASCT after CPI-based salvage regimens.

In this report, we describe three cases of ASCT performed in patients with relapsed or refractory cHL who received anti-PD-1 therapy as part of their salvage treatment. Two of the three patients developed severe ES during neutrophil recovery, one of whom required high-dose corticosteroid pulse therapy due to progressive respiratory symptoms. ES was diagnosed retrospectively based on the Spitzer and Maiolino criteria, which define ES as the occurrence of non-infectious fever, erythematous rash, pulmonary infiltrates or hypoxia, weight gain, and liver or renal dysfunction during neutrophil recovery in the absence of documented infection [15, 16]. In addition, we also investigated the dynamics of peripheral lymphocyte recovery, considering the possibility that persistent immune activation following CPI may have contributed to the inflammatory manifestations observed during engraftment.

Our findings support prior observations suggesting that checkpoint inhibition may increase the risk of engraftment-related complications, even in the autologous transplant setting. As CPIs continue to be integrated into cHL treatment algorithms, clinicians should remain vigilant for atypical post-transplant toxicities, including severe ES.

## Case presentation

### Case 1

**Fig. 1:**
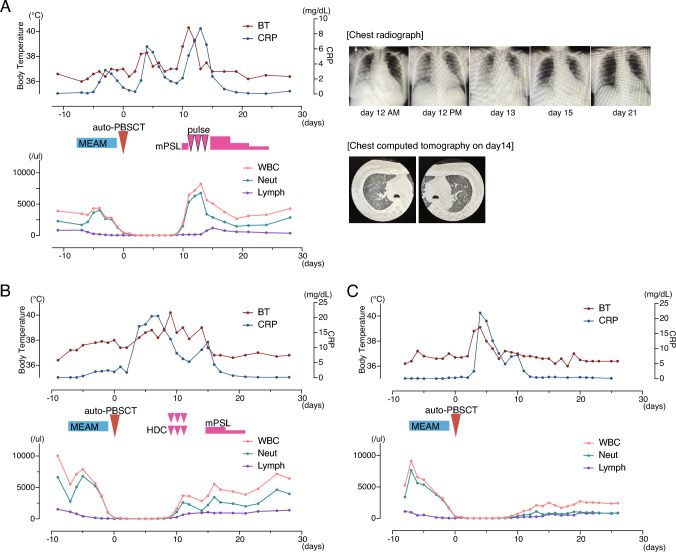
Clinical courses of the three cases following autologous peripheral blood stem cell transplantation (auto-PBSCT). Case 1 **A** developed severe ES requiring steroid pulse therapy. Case 2 **B** experienced mild ES managed with corticosteroids. Case 3 **C** had no significant ES

A 60-year-old female with cHL, nodular sclerosis (NS) type, achieved complete remission (CR) after six cycles of A + AVD [brentuximab vedotin (1.2 mg/kg), doxorubicin (25 mg/m^2^), vinblastine (6 mg/m^2^, max 10 mg), and dacarbazine (375 mg/m^2^)] as first-line therapy. However, she experienced an early relapse 2 months after completing treatment. Nivolumab (240 mg every two weeks) was initiated as salvage therapy, with no immune-related adverse events (irAEs) observed. After six doses, only partial remission (PR) was achieved. Although an allogeneic transplant was considered at this point, the patient was deemed a better candidate for autologous transplantation given her age and the anticipated risks of allogeneic hematopoietic stem cell transplantation. Subsequently, two cycles of nivolumab plus ICE [ifosfamide (5000 mg/m^2^), carboplatin (AUC 5, max 800 mg), and etoposide (100 mg/m^2^)] were administered [17]. Peripheral blood stem cells were collected after the first cycle. Following the second cycle, the patient underwent autologous peripheral blood stem cell transplantation (PBSCT) after conditioning with the MEAM regimen, a Japan-adapted variant of BEAM in which carmustine is replaced by ranimustine (MCNU) [18]. The regimen consisted of MCNU 300 mg/m^2^ on day –8, etoposide 200 mg/m^2^ on days –7 to –4, cytarabine 200 mg/m^2^ twice daily on days –7 to –4, and melphalan 70 mg/m^2^ on days –2 and –1. On day 0, a total of 6.3 × 10⁶ CD34+ autologous peripheral blood stem cells/kg were infused. Neutrophil engraftment was achieved on day +10. On day +11, the patient developed progressive hypoxia requiring 2–5 L of supplemental oxygen via nasal cannula. Inflammatory markers were elevated (CRP ~ 8 mg/dL), and no infectious cause was identified, raising suspicion for ES. Serial chest radiographs from day +12 demonstrated progressive bilateral pulmonary opacities, and a computed tomography (CT) scan on day +14 revealed bilateral ground-glass opacities with interlobular septal thickening, consistent with non-cardiogenic pulmonary edema. Representative images from both modalities are shown in Fig. 1A. Methylprednisolone (mPSL) at 1 mg/kg/day was initiated on day +12 but escalated to pulse therapy (mPSL 1 g/day for 3 days) due to worsening hypoxia (requiring 10 L via reservoir mask). The patient responded well to treatment, with rapid clinical improvement. Following the pulse therapy, mPSL was tapered from 1 mg/kg/day and discontinued by day +21. The patient remained asymptomatic during and after tapering, without recurrence of respiratory symptoms or other immune-related complications. The clinical course is illustrated in Fig. 1A, and detailed clinical parameters are provided in Supplementary Table (Table 1).

**Table 1:** Clinical characteristics, treatment timeline, and lymphocyte counts

	Case1	Case2	Case3
Age	60	41	35
Sex	Female	Male	Female
Disease type	cHL, NS	cHL, NS	cHL, NS
Prior treatment	A + AVD	A + AVD	A + AVD
Salvage treatment	Nivorumab, Nivorumab + ICE	Pembrolizumab, Nivorumab + ICE, Irradiation	Pembrolizumab
Days from last CPI to PBSCH	20	91	64
Numbers of lymphocytes at PBSCH (/μL)	534.5	2084.6	2882.3
Disease status at auto-PBSCT	PR	CR	CR
Numbers of CD34+ cells (× 10^6/kg)	6.3	3	2.7
Days from last CPI to auto-PBSCT	20	57	91
Day of neutrophil engraftment	10	10	11
Numbers of lymphocytes at engraftment (/μL)	133.2	260.8	231.3

### Case 2

A 41-year-old male with cHL, NS type, initially received six cycles of A + AVD, achieving PR. He subsequently underwent involved-field radiotherapy to the residual lesion, resulting in CR. Six months later, disease relapse occurred. Pembrolizumab (200 mg every 3 weeks) was administered for 10 cycles as salvage therapy, without any irAEs or significant toxicity. Additional radiotherapy was performed, and metabolic CR was achieved. Stem cell collection was performed at this time. However, another relapse was detected prior to transplant. Two cycles of nivolumab plus ICE were administered, resulting in complete remission. Conditioning with the MEAM regimen was followed by PBSCT. On day 0, a total of 6.3 × 10⁶ CD34+ autologous peripheral blood stem cells/kg were infused. Neutrophil engraftment occurred on day + 10. On day + 9, the patient developed transient fever suggestive of early ES, which was managed conservatively with intermittent hydrocortisone. On day + 14, he developed an erythematous skin rash and mild hypoxia. Intravenous mPSL at 1 mg/kg/day was initiated, leading to the prompt resolution of symptoms. No further complications occurred. The clinical course is presented in Fig. 1B and Supplementary Table.

### Case 3

A 35-year-old female with cHL, NS type, achieved CR after six cycles of A + AVD as first-line therapy. Six months after treatment completion, relapse was detected. Pembrolizumab (200 mg every 3 weeks) was administered as salvage therapy for six cycles, achieving complete metabolic remission. Stem cell collection was successfully performed thereafter. She underwent high-dose conditioning with the MEAM regimen, followed by PBSCT. On day 0, a total of 2.74 × 10⁶ CD34⁺ autologous peripheral blood stem cells/kg were infused. Neutrophil engraftment was achieved on day + 11. Around day + 10, the patient developed transient fever (> 38 °C) accompanied by a spike in CRP to over 20 mg/dL; however, there were no accompanying signs such as hypoxia, rash, or edema. No corticosteroids were required, and the post-transplant course was uneventful. The clinical timeline is depicted in Fig. 1C, and Supplementary Table.

### Analysis of lymphocyte counts and CPI-to-transplant intervals

Given that CPIs may influence immune cell kinetics beyond their period of administration, we investigated their potential effects on lymphocyte dynamics and the timing of transplantation. Specifically, we analyzed the absolute lymphocyte counts at the time of peripheral blood stem cell harvest (PBSCH) and during the engraftment phase, as well as the intervals from the last CPI dose to both PBSCH and autologous peripheral blood stem cell transplantation (auto-PBSCT). Absolute lymphocyte counts at PBSCH were 534.5/μL in Case 1, 2084.6/μL in Case 2, and 2882.3/μL in Case 3. At the time of neutrophil engraftment, lymphocyte counts were 133.2/μL in Case 1, 260.8/μL in Case 2, and 231.3/μL in Case 3. The interval from the final CPI administration to PBSCH was 20 days in Case 1, 64 days in Case 3, and 91 days in Case 2, while the corresponding interval to auto-PBSCT was 20, 91, and 57 days, respectively. Notably, both intervals were shortest in Case 1, who experienced the most severe ES.

## Discussion

This case series highlights a relatively under-recognized clinical concern: the occurrence of ES following ASCT in patients with cHL previously treated with CPIs. While prior studies have suggested an increased incidence of ES in this setting, few have examined accompanying immune parameters in detail. By analyzing lymphocyte kinetics and the temporal relationship between CPI exposure and transplantation, our report adds to the emerging clinical awareness regarding CPI-associated inflammatory complications in the autologous transplant setting.

Among the three cases presented, two patients developed ES during neutrophil recovery. One case (Case 1) experienced severe respiratory symptoms requiring high-dose corticosteroid pulse therapy, while the other (Case 2) exhibited a milder presentation that resolved with moderate-dose corticosteroids. The third patient (Case 3) had only a transient fever and recovered without immunosuppressive treatment. Despite its severity, ES in Case 1 responded well to corticosteroids, suggesting that CPI exposure does not preclude steroid responsiveness. These outcomes align with previous retrospective reports describing variable ES severity in cHL patients who received CPIs prior to ASCT [13, 14].

We explored whether lymphocyte-related parameters might be associated with ES development. Although absolute lymphocyte counts at the time of stem cell harvest and engraftment differed markedly among patients, no consistent correlation with ES severity was evident. Interestingly, despite having the lowest lymphocyte counts at both harvest and engraftment, Case 1 developed the most severe ES. These findings suggest that lymphocyte quantity alone may not serve as a reliable predictor of ES risk.

In contrast, the timing of transplantation relative to CPI exposure may be more relevant. Case 1, who experienced the most severe ES, had the shortest interval (20 days) from the final CPI dose to both stem cell collection and transplantation. Case 3, who remained asymptomatic, had the longest CPI-free intervals (64 and 91 days, respectively). Although based on limited numbers, this pattern raises the possibility that shorter CPI-to-transplant intervals may contribute to heightened inflammatory responses during engraftment. This hypothesis is supported by prior studies indicating that CPI induces sustained immunologic effects that can persist for several weeks or longer after treatment discontinuation [19]. Persistent T-cell activation and altered cytokine profiles may shape the immune milieu at the time of neutrophil recovery, potentially predisposing to inflammatory toxicities such as ES, even in the absence of allogeneic immunity.

In clinical practice, as demonstrated by Case 1, it is often challenging to secure a sufficient wash-out period between the last dose of CPI and ASCT due to disease progression or transplant logistics. Moreover, “atypical post-transplant immune reactions” may include non-infectious fever, mild respiratory symptoms, or skin rash without meeting full diagnostic criteria for ES. Given the potential for exaggerated immune responses in CPI-exposed patients, clinicians should maintain a high index of suspicion and consider early corticosteroid intervention, even in borderline or atypical presentations.

As CPI-based salvage regimens become increasingly common in cHL, awareness of their potential effects on transplant-related inflammation is essential. Clinicians should consider the timing of ASCT in relation to CPI administration and monitor for atypical post-transplant immune reactions. Prospective studies will be needed to validate these findings, define optimal transplant timing, and identify biomarkers to stratify the risk of ES in this emerging clinical context.

## Supplementary Information

Below is the link to the electronic supplementary material.

Supplementary file1 (XLSX 15 KB)

## Data Availability

The datasets generated during and/or analyzed in the current study are available from the corresponding author on reasonable request. However, access to patient-level data is subject to ethical and legal restrictions.
